# Effects of the Anaerobic Respiration of *Shewanella oneidensis* MR-1 on the Stability of Extracellular U(VI) Nanofibers

**DOI:** 10.1264/jsme2.ME12149

**Published:** 2013-05-29

**Authors:** Shenghua Jiang, Hor-Gil Hur

**Affiliations:** 1 School of Environmental Science and Engineering, Gwangju Institute of Science and Technology, Gwangju 500–712, Republic of Korea; 2 International Environmental Analysis and Education Center, Gwangju Institute of Science and Technology, Gwangju 500–712, Republic of Korea

**Keywords:** *Shewanella*, uranium, nanofibers, nanoparticles, mutant

## Abstract

Uranium (VI) is considered to be one of the most widely dispersed and problematic environmental contaminants, due in large part to its high solubility and great mobility in natural aquatic systems. We previously reported that under anaerobic conditions, *Shewanella oneidensis* MR-1 grown in medium containing uranyl acetate rapidly accumulated long, extracellular, ultrafine U(VI) nanofibers composed of polycrystalline chains of discrete meta-schoepite (UO^3^·2H_2_O) nanocrystallites. Wild-type MR-1 finally transformed the uranium (VI) nanofibers to uranium (IV) nanoparticles via further reduction. In order to investigate the influence of the respiratory chain in the uranium transformation process, a series of mutant strains lacking a periplasmic cytochrome MtrA, outer membrane (OM) cytochrome MtrC and OmcA, a tetraheme cytochrome CymA anchored to the cytoplasmic membrane, and a trans-OM protein MtrB, were tested in this study. Although all the mutants produced U(VI) nanofibers like the wild type, the transformation rates from U(VI) nanofibers to U(IV) nanoparticles varied; in particular, the mutant with deletion in tetraheme cytochrome CymA stably maintained the uranium (VI) nanofibers, suggesting that the respiratory chain of *S. oneidensis* MR-1 is probably involved in the stability of extracellular U(VI) nanofibers, which might be easily treated via the physical processes of filtration or flocculation for the remediation of uranium contamination in sediments and aquifers, as well as the recovery of uranium in manufacturing processes.

Uranium is a radioactive and chemically toxic element that is widespread in the environment, primarily due to anthropogenic activity (25). Hexavalent uranium (VI) is considered to be one of the most widely dispersed and problematic environmental contaminants, due in large part to its high solubility and great mobility in natural aquatic systems (9). Investigations concerning the mineralization and transformation of hexavalent uranium complexes are of critical importance in terms of the long-term management of spent nuclear fuels, the remediation of uranium-contaminated environments and the recovery of uranium minerals. The immobilization of uranium, primarily through the reduction of uranium (VI) to uranium (IV), which has relatively lower solubility and mobility, has long been thought a viable remediation strategy.

Due to its marked respiratory versatility, *Shewanella* sp. strains have received more recent attention since they are capable of reducing iron (III), uranium (VI), technetium (VII), and many other metals (3, 7–9, 15, 27). The product of bacterial uranium reduction is commonly reported to be tetravalent uraninite (UO_2_) nanoparticles, with subtly different morphologies, that are located either outside the cell or in the periplasm (10, 21). Although the bacterial reduction of U(VI) and immobilization of U(IV) as uraninite nanoparticles has been well studied in recent years, the deposition of uranium (VI) by microorganisms is poorly understood The uranium (VI) mineral schoepite ([UO_2_]_8_O_2_[OH]_12_)(H_2_O)_12_ or UO_3_·2.25H_2_O was first described in 1923 (24). While a series of related minerals, including meta-schoepite was obtained through a variety of chemical routes, and their structure and composition have been characterized (5, 19, 26), the biogenic formation of uranium (VI) minerals remains rarely investigated. Considering the 1-dimensional morphology and physicochemical properties, hexavalent uranium nanofibers could be the preferred form for uranium bio-remediation rather than the 0-dimensional U(IV) nanoparticles. We previously reported that wild-type *S. oneidensis* MR-1 produces extracellular uranium (VI) nanofibers; however, the U(VI) nanofibers finally transformed to U(IV) nanoparticles via the complete reduction of U(VI) (6).

*S. oneidensis* MR-1 contains 42 putative *c*-type cytochromes (1, 10) and mutagenesis studies have shown that some of them are essential for metal reduction. Among these, periplasmic, decaheme, cytochrome (MtrA), decaheme cytochromes (MtrC and OmcA) exposed on the outer membrane (OM), and a tetraheme cytochrome (CymA) anchored to the cytoplasmic membrane, have all been known to be required for uranium reduction (3, 10, 22). In addition, a trans-OM protein MtrB, as part of a membrane-spanning protein complex (MtrABC), is needed for uranium reduction (4, 18). In order to investigate the respiratory activities influencing U(VI) reduction and further transformation, a series of mutants lacking CymA, MtrA, MtrB, or both MtrC and OmcA were tested in this study for the controllable formation of extracellular uranium (VI) nanofibers.

## Materials and Methods

### Bacterial growth conditions

*Shewanella* strains used in this study were *S. oneidensis* MR-1, and a series of mutants lacking either *cym*A, *mtr*A, *mtr*B, or both *mtr*C and *omc*A genes (2, 3, 10, 14, 16, 22). The mutant strains of *S. oneidensis* MR-1 were gifts from Dr. James K. Fredrickson in Pacific Northwest National Laboratory, Richland, USA. The culture medium and incubation conditions were in accordance with our previous study (6). Briefly, the culture media contained 30 mM NaHCO_3_, 10 mM sodium dl-lactate as the electron donor, and ~2 mM uranyl acetate (UO_2_[CH_3_COO]_2_·2H_2_O) as an electron accepter for the synthesis of uranium nanofibers. The pH value of the medium was adjusted to 7.0. The *Shewanella* strains were inoculated into sealed serum bottles with the 30 mL N_2_ purged culture medium at a final cell density of 2×10^8^ cells mL^−1^. All the cultures were incubated anaerobically in the dark at 30°C for 120 h.

### Measurements of U(VI) and lactate in the culture media

The samples were collected at selected times during incubation for the detection of soluble uranium and lactate consumption in aqueous medium. To measure the concentration of uranium, the culture supernatants were filtered through a 0.2 μm membrane filter (MFS-25; Advantec MFS, Dublin, CA), and the filtrates were diluted and acidified with 2% HNO_3_ for analysis using inductively-coupled plasma mass spectrometry (7500ce, ICP-MS; Agilent Technologies, Palo Alto, CA). The concentrations of the organic acids were detected by HPLC (Shimazu, Tokyo, Japan), which was equipped with a SPD-10A UV detector (Shimazu) and a Shodex RSpak KC-811 (8.0 mm ID*300 mm) column (Shodex, Tokyo, Japan). The mobile phase was 5 mM sulfuric acid with a flow rate of 0.5 mL min^−1^, and UV detection was performed at 210 nm.

### Characterization of materials

Transmission electron microscopic (TEM) was performed with samples collected to observe the mineralogical morphologies. To prepare the electron microscopy specimen, the precipitates in the bacterial culture media were collected and washed with DI water three times. Such specimens were then dried on a Cu grid under ambient conditions for TEM (TEM; JEOL, Tokyo, Japan).

For mineralogical analysis, the minerals produced by bacteria were collected from culture bottles with syringes after incubation and washed with DI water three times by centrifugation at 2,300×*g* for 10 min (5415D; Eppendorf, Hamburg, Germany). The washed minerals were dried under anaerobic conditions in a glove box. X-ray diffraction (XRD) analysis was performed using a Rigaku D/MAX Ultima III high resolution X-ray diffractometer (Rigaku, Tokyo, Japan) with Cu K irradiation. The generator was operated at 40 kV and 40 mA.

## Results and Discussion

### Formation of U(VI) nanofibers and U(IV) nanoparticles

**Fig. 1:**
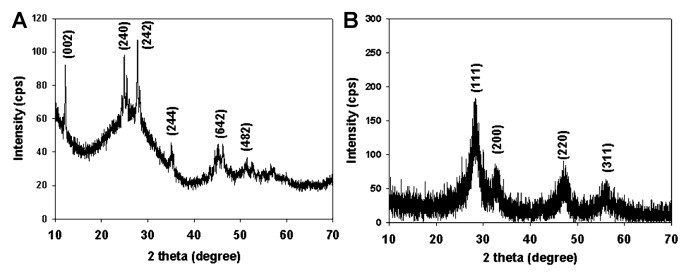
XRD analyses of U(VI) nanofibers formed by CymA- mutant (A) and U(IV) nanoparticles formed by WT (B) after 120 h incubation. Peaks in the diffraction pattern are labeled with Miller indices, *h k l*, indicating the set of lattice planes responsible for the diffraction peak.

**Fig. 2:**
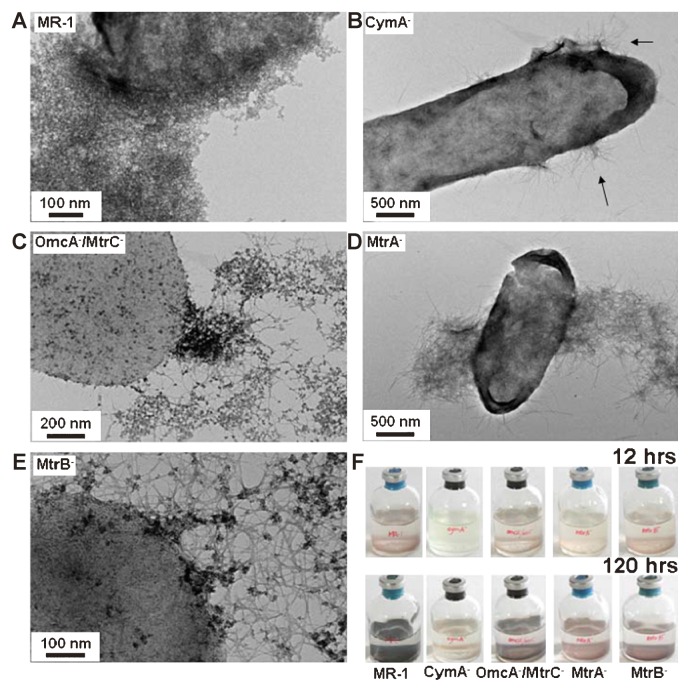
TEM images of the uranium nanoparticles and nanofibers (arrows) formed by wild-type *S. oneidensis* MR-1 (A), and mutants of CymA^−^ (B), MtrC^−^/OmcA^−^ (C), MtrA^−^ (D), and MtrB^−^ (E) after 120 h incubation. The color change of the precipitate by the wild type and mutants after 12 and 120 h incubation is shown (F).

After 12 h incubation, brown flocculent precipitate formed first by the wild-type strain. TEM images showed that filamentous structures, approximately 1–4 nm in width appeared in the bacterial cultures, as in our previous report (Fig. S1 and S2). Our previous cryo-EM analysis confirmed that the filaments accumulated on the cell surface under in situ conditions rather than being artificially formed during sample preparation before electron microscopy (6). Results of XRD analysis of uranium nanofibers produced by wild-type MR-1 was compared with the data from the Powder Diffraction File (PDF #43-0364), and indicated that the mineral was likely meta-schoepite (UO_3_·2H_2_O, orthorhombic) (6). As the incubation time proceeded, the nanofibers were further transformed into particulate shapes in the sample collected at 120 h (Fig. S3) and XRD patterns indicated that the nanoparticles were composed of tetravalent uraninite (UO_2_), as previously reported (10, 20, 21, 23) (Fig. 1B). In contrast, although the mutants with deletions in MtrC/OmcA, MtrA, MtrB or CymA also formed similar uranium nanofibers at an early stage, the final products produced by the former three mutants were a mixture of U(VI) nanofibers and U(IV) nanoparticles. The CymA^−^ mutant stably maintained U(VI) nanofibers up to 120 h of incubation (Fig. 2). The results of XRD analysis confirmed that the final product at 120 h in the culture with CymA^−^ mutant was likely meta-schoepite (Fig. 1A).

### Mechanisms of the transformation of U(VI) nanofibers to U(IV) nanoparticles

**Fig. 3:**
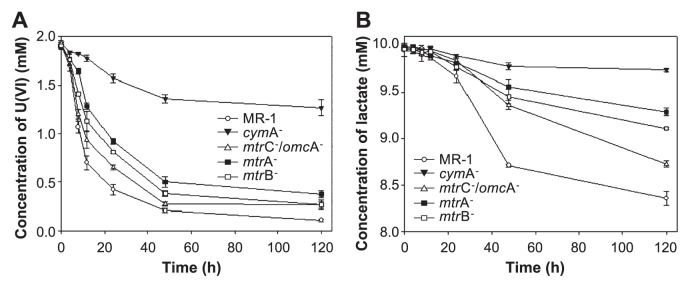
Consumption of the electron acceptor uranium (VI), and lactate, the electron donor. (A) The time-dependent concentration of soluble uranium (VI), and (B) lactate remaining in culture media inoculated with wild-type and mutant strains under anaerobic conditions. Data points from three parallel independent incubations.

Kinetic analyses indicated that 70% of the soluble uranium in the culture medium of wild-type *S. oneidensis* MR-1 rapidly decreased over 12 h. Similarly, the concentration of soluble uranium in culture media inoculated with the MtrC^−^/OmcA^−^, MtrB^−^ and MtrA^−^ mutants decreased by 50, 40 and 30% after 12 h, respectively. In contrast, <10% of uranium (VI) was removed after 12 h in culture medium inoculated with the CymA^−^ mutant, which appears to be blocked in electron transport (3) (Fig. 3A).

The consumption of the electron acceptor uranium (VI), and the electron donor lactate by the strains was not stoichiometric, especially during the first 12 h of incubation (Fig. 3). However, after the 12 h incubation period, lactate consumption by the wild-type strain noticeably increased as the medium color changed from brown to black, indicating that lactate consumption by strain MR-1 was coupled to the reduction of uranium (VI) to uranium (IV), rather than the accumulation of uranium (VI) nanofibers at an early stage. The mutant strains tested in this study also showed a similar phenomenon, although less U(VI) were precipitated from the culture solution than was seen with the wild-type strain (Fig. 3). These results suggested that the accumulation of uranium (VI) nanofibers is not predominantly associated with U(VI) reduction by lactate consumption. The different rates of soluble uranium (VI) decrease and uranium (VI) nanofiber formation by the wild type and mutants might be due to different amounts of the initially-formed U(IV) nanoparticles on the cell surface, which acts as a nucleus for the growth of U(VI) nanofibers.

The normalized U K-edge X-ray absorption near edge structure (XANES) spectra obtained in our previous study (6) suggested the transformation of the uranium nanostructures by wild-type MR-1 involving several steps as follows: 1) initial absorption of uranium (VI) to bacterial cell surfaces, 2) fast dynamic reduction of a small amount of uranium (VI) to uranium (IV), 3) accumulation of UO_2_ precipitates on the bacterial cell surfaces and/or in the periplasm that appear to play a role in triggering the formation of extracellular uranium (VI) nanofibers, and 4) reduction of the uranium (VI) nanofibers to uranium (IV) nanoparticles with the concurrent consumption of lactate.

The mutants also showed a similar process but the accumulated U(VI) nanofibers were not finally reduced to U(IV) nanoparticles (Fig. 2). Although the mutants lack various cytochromes or outer membrane protein involved in metal reduction, slow, but continuous reduction of uranium occurred by the mutant strains. Alternative proteins or other unknown factors likely active and participate in a low level of activity that is required for initial reduction of soluble U(VI) to U(IV) nanoparticles on the cell surface, which acted as a nucleus for the growth and accumulation of U(VI) nanofibers. Under a defined set of conditions, the U(VI) nanofibers appeared to be formed and accumulated as long as the minimum amount of U(IV) formed by either wild-type MR-1 or the mutants as a nucleus on the cell surface at the initial stage, regardless of the further reduction of the formed U(VI) nanofibers. The initially minimum reduction of U(VI) might be due to the activities of alternative cytochromes or other unknown factors. However, after the U(VI) nanofibers formed and accumulated, the mutant strains showed much less transformation of U(VI) to U(IV) than that by the wild-type strain. It should be noted that the CymA^−^ mutant, which appeared to be disabled from the further reduction of insoluble U(VI) fibers to insoluble U(IV) nanoparticles, can stably maintains U(VI) nanofibers. However, it has been reported that the CymA^−^ mutant did not completely abolish U(VI)-reducing activity due to the presence of possible multiple pathways for soluble U(VI) reduction (3). This discrepancy in U(VI) reduction by the CymA^−^ mutant is probably due to the blockage of alternative pathways or factors affecting electron transfer to extracellular solid U(VI) nanofibers. The results obtained in this study strongly suggested that the cytochromes and OM proteins significantly influence uranium reduction and the formation of U(VI) nanofibers, and subsequent further transformation to U(IV) nanoparticles.

### Environmental implication

The contamination of surface and ground water with uranium is a serious environmental concern. Results of the current study suggest that it may be possible to immobilize soluble uranium (VI) into uranium (VI) nanofibers by using facultatively anaerobic *Shewanella* strains and a series of mutants. Although the biogenic production of uraninite (UO_2_) nanoparticles has previously been suggested as a remediation strategy to remove soluble uranium (VI) from the environment, this mineral form is likely to be mobile in porous sediments and rapidly re-oxidized due to its nanometer-scale size (12, 17, 23). In contrast, 1-dimensional uranium (VI) nanofibers may offer another method for the removal of soluble uranium (VI) from the environment via physical processes of filtration and flocculation. The mutant strains of *S. oneidensis* MR-1, especially CymA^−^, allows the synthesis and stabilization of unique, long, and ultrafine uranium (VI) nanofibers, which are distinct from previously reported biogenic tetravalent uraninite nanoparticles. The biological transformation of uranium (VI) nanofibers may also provide an alternative biological tool for “Yellow Cake” manufacturing processes, which currently use diverse harsh physicochemical treatments to extract and leach uranium from ores (11, 13).

## Supplementary Material
