# Supplementary material for: Effects of the Anaerobic Respiration of Shewanella oneidensis MR-1 on the Stability of Extracellular U(VI) Nanofibers
Source: Microbes Environ. 2013 May 29;28(3):312–5. doi: 10.1264/jsme2.ME12149 (PMC4070970; doi:10.1264/jsme2.ME12149)
Supplement: Supplementary file 1 [file 28_312_s1.pdf]

Supplementary Material for

Effects of the Anaerobic Respiration of *Shewanella Oneidensis* MR-1 on the Stability of Extracellular U(VI) Nanofibers

Shenghua Jiang[1,2], and Hor-Gil Hur[1*]

[1]School of Environmental Science and Engineering, and [2]International Environmental Analysis and Education Center, Gwangju Institute of Science and Technology, Gwangju 500-712, Republic of Korea.

[**]Corresponding author

Hor-Gil Hur

Tel: +82-62-970-2437; Fax: +82-62-970-2434; E-mail: hghur@gist.ac.kr

Fig. S1

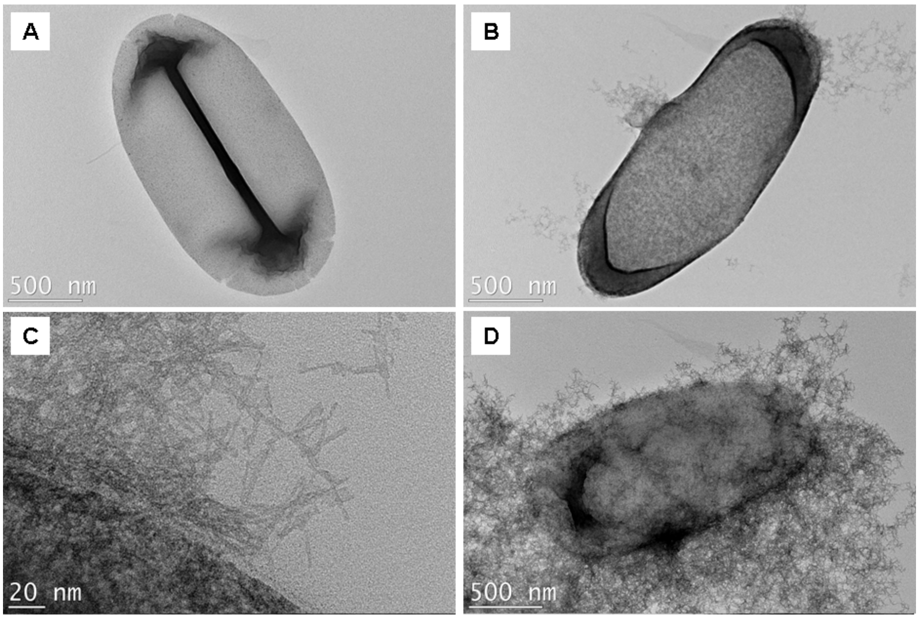

Fig. S1. TEM images showing formation of U(VI) nanofibers by wild type *S. oneidensis* MR-1 taken at (A) 0 h, (B and C) 8h, and (D) 12 h incubation.

Fig. S2

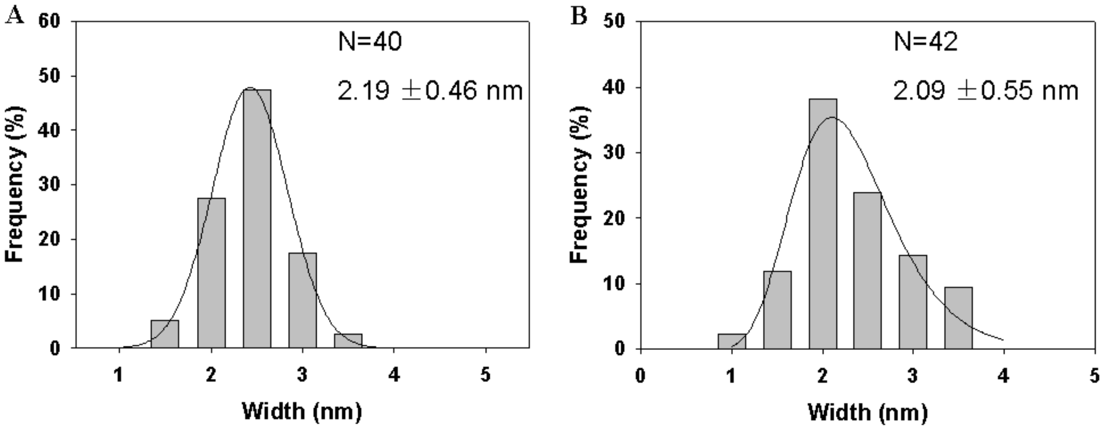

Fig. S2. Size distribution of U(VI) nanofibers (A) and U(IV) nanoparticles (B) formed by wild type *S. oneidensis* MR-1. Numbers of the nanofibers and nanoparticles counted, average, and standard deviations of the width of the U(VI) nanostructures are shown on the diagram. Solid lines: Estimation by log normal function.

Fig. S3

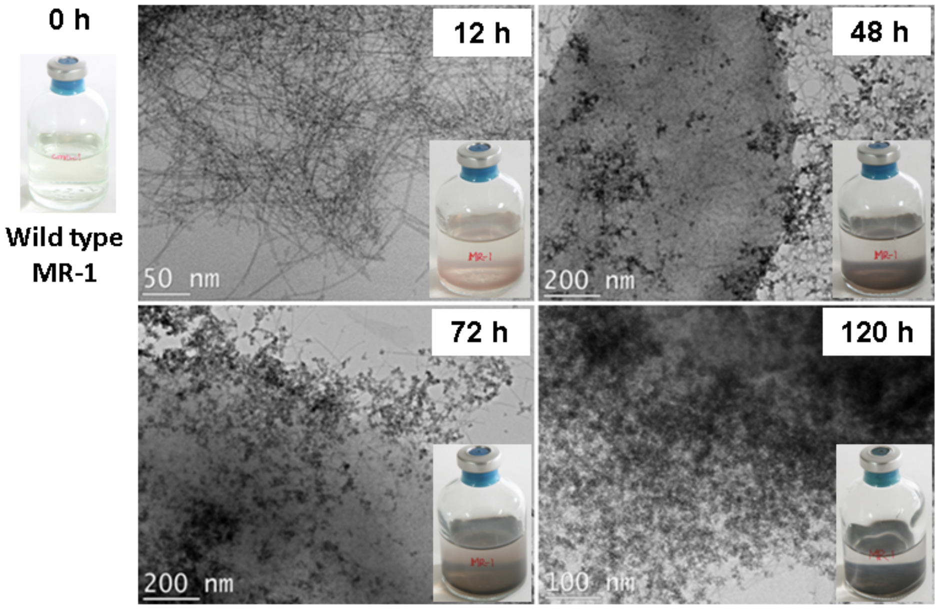

Fig. S3. TEM images showing the transformation of U(VI) nanofibers to U(IV) nanoparticles by wild type *S. oneidensis* MR-1. Color changes of the precipitate formed in the culture medium with incubation time is also shown.
